# Granulomatosis with polyangiitis: clinical characteristics and updates in diagnosis

**DOI:** 10.3389/fmed.2024.1369233

**Published:** 2024-08-27

**Authors:** Malgorzata Potentas-Policewicz, Justyna Fijolek

**Affiliations:** ^1^Department of Geriatrics, Dr Anna Gostynska Wolski Hospital, Warsaw, Poland; ^2^The Third Department of Pneumonology and Oncology, National Tuberculosis and Lung Diseases Research Institute, Warsaw, Poland

**Keywords:** anti-neutrophil cytoplasmic antibodies, vasculitis, granulomatosis with polyangiitis, inflammation, diagnostic techniques, leukocyte proteinase 3, myeloperoxidase

## Abstract

Granulomatosis with polyangiitis (GPA) is a rare systemic disease characterized by granulomatous inflammation of the respiratory tract and necrotizing vasculitis of small and medium vessels often associated with the production of anti-neutrophil cytoplasmic antibodies (ANCA) directed mainly against leukocyte proteinase 3 (PR3). Usually, it involves upper airways, lungs, and kidneys, however any organ may be affected. The diagnosis is based on clinical, radiological, and serological findings. Biopsies, although strongly recommended, are not always feasible and often provides non-specific features. ANCA plays a crucial role in the diagnosis of GPA; nevertheless, ANCA detection is not a substitute for biopsy, which plays an important role in suspected cases, particularly when histological confirmation cannot be obtained. Significant advances have been made in classification criteria and phenotyping of the disease, particularly in determining the nuances between PR3-ANCA and myeloperoxidase (MPO)-ANCA vasculitis. This has led to better characterization of patients and the development of targeted treatment in the future. In addition, better identification of cytokine and immunological profiles may result in immuno-phenotyping becoming a new approach to identify patients with ANCA-associated vasculitis (AAV). Due to the chronic relapsing–remitting nature, strict follow-up of GPA is necessary to provide appropriate management. The search for the accurate marker of disease activity and to predict relapse is still ongoing and no predictor has been found to reliably guide therapeutic decision-making.

## 1 Introduction

Granulomatosis with polyangiitis (GPA; formerly known as Wegener’s granulomatosis) is a rare necrotizing vasculitis combining inflammation of the vascular wall and peri- and extravascular granulomatosis (1). According to current nomenclature classification it belongs to the anti-neutrophil cytoplasmic antibody (ANCA)-associated vasculitis (AAV) group, alongside microscopic polyangiitis (MPA) and eosinophilic granulomatosis with polyangiitis (EGPA) (2). Each of these conditions is associated with a circulating ANCA targeted mainly against leukocyte proteinase 3 (PR3), and myeloperoxidase (MPO). Patients with GPA tend to be PR3-ANCA positive, while those with MPA and EGPA are predominantly MPO-ANCA positive (3).

AAV is a group of rare diseases with an estimated prevalence of 200–400 cases per million people. Although there was an increase in incidence over time, this is likely due to increased awareness of the diagnosis and yield in ANCA testing methodologies (4). In the recent study from Sweden the incidence of AAV was stable over the course of 23 years (15.4/million), while the prevalence markedly increased due to improved management and survival (5). Among all three types of AAV, GPA is the most common with an average annual incidence/million adults from 7.7 to 15.4 in European countries (5, 6). The peak age at diagnosis varies, depending on the study population, such as 45–55 years (in an Italian cohort) (7) and 65–74 years (in the UK) (8). However, a shift in the peak age at onset toward the higher age range between the groups has been observed, during the last two-three decades (5). AAV may also occur in pediatric populations but epidemiological data are scarce and poorly characterized. Childhood primary vasculitis is diagnosed in 3% of all children referred to pediatric rheumatology departments, while the incidence of GPA ranges from 0.88 to 1.8 per million individuals (9).

GPA typically involves the upper respiratory tract, lungs, and kidneys (acronym ELK); however, any organ can be involved. The spectrum and severity of the disease are heterogeneous, ranging from indolent disease involving only one site to fulminant, multiorgan vasculitis leading to death (10). The variety and non-specificity of symptoms often contribute to diagnostic delays. In a report of 912 patients with GPA, the time from initial symptoms to diagnosis varied greatly, from one month to over three years, with 36% of all symptoms reported more than one year prior to diagnosis (11).

To date, diagnostic criteria are established neither for GPA nor for special diagnostic tests. Therefore, diagnosis is mostly based on clinical features, in combination with laboratory and imaging findings; it may be supported by the presence of ANCA and histological examination. Nonetheless, in recent decades, notable progress has been made in understanding the disease and development of diagnostic techniques, particularly imaging modalities, thus contributing to early diagnosis and early implementation of appropriate treatment to avoid essential organ damage. In the present study, we review the clinical features of GPA (focusing on pulmonary symptoms) and updates in diagnosis, considering disease assessment and monitoring.

## 2 Pathogenesis

**Figure 1:**
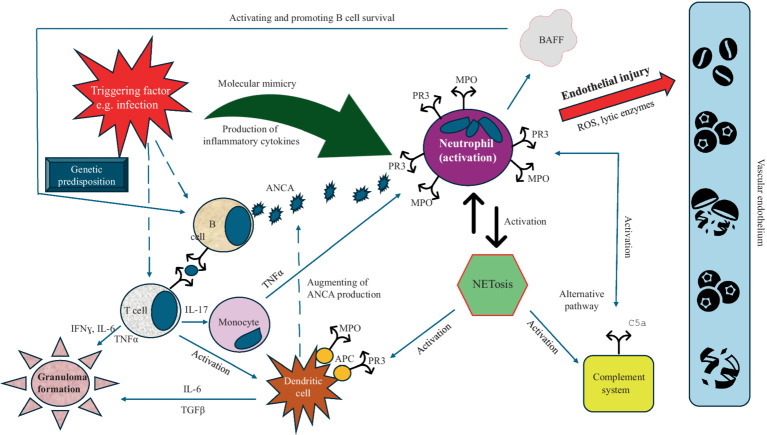
Pathogenesis of GPA. Some trigger (e.g., infection) to genetically predisposed subject stimulates neutrophils and B and T cells. As a result of neutrophil activation, PR3 and MPO are expressed and translocate into the cytoplasmic membrane and the production of BAFF increases, which promotes B cells survival and stimulates them to produce ANCA. These antibodies bind to PR3 and/or MPO and activate neutrophils, which subsequently accumulate at the wall of the vessel and release reactive oxygen species and proteases leading to endothelium injury and necrosis. The activation of neutrophils results in the NETs formation, which, in turn, augment the production of ANCA by stimulating (together with T cells) of the dendritic cells. NETs activate also the complement system that additionally stimulates neutrophils through the release of neutrophil-activating chemokines (e.g., C5a). In addition, the abnormal T and dendritic cells cytokine response provides help to B cells for the production ANCA and granulomas formation (Abbreviations: PR3, proteinase 3; MPO, myeloperoxidase; BAFF, B-cell activating factor; ROS, reactive oxygen species; ANCA, antineutrophil cytoplasmic antibody; NETs, neutrophil extracellular traps; TNFα, tumor necrosis factor α; IL, interleukin; TGFβ, transforming growth factor β; IFNɣ, interferon ɣ).

The pathogenesis of GPA is complex and multifactorial, with ANCA, neutrophils, B and T lymphocytes, monocytes, endothelial cells, and the alternative complement pathway playing important roles (12, 13). The pathogenic hallmark is the loss of immunological T and B cell tolerance to neutrophilic proteins, namely PR3 or MPO (12). This process occurs alongside risk factors such as genetic background, age, environmental factors, and inflammation and/or infection (Figure 1) (14, 15). Interestingly, there are reports that GPA may arise from a similar genetic predisposition as rheumatoid arthritis (RA) (16). Therefore, a rare case of two family members with GPA highlights the potential genetic underpinnings of this disease (17).

## 3 Clinical features

The classic form of GPA involves upper airway (ear, nose, throat; ENT), lung, and kidney involvement; however, any organ can be affected. Constitutional symptoms, such as myalgias, loss of appetite, weight loss, fatigue, fever, night sweats, and migratory arthropathy, appear in 50% of patients and may precede weeks to months prior to the onset of clinically apparent organ-involvement (18). However, in about one third of patients, GPA begins with granulomatous inflammation of the upper respiratory tract, without general symptoms, and the presence of ANCA is detected only in a part of such patients. This phase may last for variable times and the disease at this stage is hard to recognize (19). More often, GPA is a rapidly progressive disease from the onset, with systemic presentation; however, the findings that should raise suspicion for this vasculitis include involvement of multiple organs.

### 3.1 Pulmonary involvement

The incidence of pulmonary involvement in GPA varies between 62 and 90%, depending on the study (20–22). The clinical symptoms are heterogeneous, ranging from asymptomatic cases through non-specific signs as cough, dyspnea, or hemoptysis, to acute respiratory failure requiring ventilatory support. In GPA, three main presentations of lung involvement can be distinguished: necrotizing granulomatous inflammation (NGI), tracheobronchial inflammation, and pulmonary capillaritis manifesting as DAH. The next rare presentation is interstitial lung disease (ILD) which occurs more frequently in MPA (23).

#### 3.1.1 NGI

The NGI is the hallmark feature defining GPA, and the main feature distinguishing GPA from MPA (2). This inflammation in the lungs manifests as nodules (single or multiple) or mass lesions being the most common lung manifestation of GPA (40–70%) (24). In 20–50% of patients, cavities are observed (25), which develop when the central necrosis of the granulomatous lesion feeds into a draining airway, and this may cause coughing and hemoptysis, and always require differentiation from infection and neoplasms (26). Other manifestations include bilateral parenchymal infiltrates and consolidations presenting in around half of the patients (27). Lung lesions related to the NGI may wax or wan spontaneously, with some nodules disappearing and others appearing over time (23). Although these patients usually respond well for corticosteroids (CS) and immunosuppressive (IS) therapy, they often have fibrotic strands as residual scars, and should not be confused with ILD, which is mostly associated with MPO-AAV (26).

#### 3.1.2 Tracheobronchial inflammation

The frequency of tracheobronchial involvement varies from 13.6–55% in patients with GPA (28, 29), and is more frequent in women and younger patients (26, 29). Tracheobronchial inflammation may occur anywhere in the tracheobronchial tree, with subglottic region is most involved in this region (26, 29). Furthermore, tracheobronchial involvement may occur at any stage of the disease, even in patients being in remission. On rare occasions, it may be the only feature of GPA, making a proper diagnosis difficult, especially in cases of ANCA negativity (28, 29). The pattern of lesions is heterogeneous, however, four main types are distinguished: mucosal abnormalities, masses and polyps, subglottic stenosis (SGS), and tracheobronchial inflammation and stenoses (TBISs). Other rarer complications include tracheo- or bronchomalacia as well as bronchiectasis (29), with the latter occurring more frequently in patients with AAV and MPO positivity (30). Generally, inflammatory processes affecting lower airways in GPA are similar to those affecting nasal mucosa; however, they are non-specific and should be differentiated from other causes (29).

Regarding mucosal abnormalities, they may occur anywhere in the tracheobronchial tree, be patchy in distribution, and occur as isolated lesions. The most common forms are mucosal edema, erythema, thickening, and granularity and/or ulceration of mucosal surface (29). Purulent secretions and excessive mucous production are also common, especially in active disease. Masses and polyps are usually a reflection of active disease. They are also referred to as inflammatory pseudotumor, and because they mimic malignancy, biopsy and histological examination is necessary (31). These lesions may ulcerate, cause cough, and hemoptysis.

SGS is the most common manifestation of tracheobronchial involvement in GPA occurring in 10–20% of patients and may occur in any stage of the disease (19). In one of the cohorts (28) SGS developed in 13.6% of patients with GPA and its frequency increased in parallel with patient survival time; furthermore, SGS was not reflective of disease activity in the organs in 44% of cases (28). Patients with SGS are more likely to be female, younger at time of diagnosis, and have saddle-nose deformities, however, are less likely to have renal involvement (32). Symptoms can be non-specific, and increase gradually, allowing the patient to adjust his breathing pattern until the critical stenotic airway area is reached (19). Patients may present with progressive dyspnea and stridor, dry cough, wheeze, and hoarseness (33). The stridor and wheeze may be confused with the wheeze of asthma, often leading to misdiagnosis, especially when there is no other organ involvement. Of note, as the airway caliber narrows, mucous plugging becomes a great concern, as it can cause acute stridulous exacerbations and airway obstruction (19). Patients with significant stenosis (approximately 80%) can present life-threatening respiratory symptoms requiring even tracheostomy (19). The diagnosis of SGS in patients with known GPA is straightforward and must be presumed, even when histology is not definitive (10). The diagnostic challenges are cases of isolated SGS, without the presence of ANCA, where the diagnosis should be based on histological examination, after exclusion of other causes (33).

TBISs are less frequent than SGS, and often associated with GPA activity elsewhere (32). The inflammation may be localized or complex involving multiple tracheal or bronchial segmental stenoses with luminal distortion; however, isolated bronchial stenosis has been described (34). The stenoses can be mild to severe, and typically can vary in their severity from one bronchus to another in the same lobe (29). Most of the severely narrowed bronchi become irreversibly occluded from fibrotic scarring (29). Rarely, complete closure of the bronchus may occur, which may even require pneumonectomy (35). Generally, symptoms of TBISs are similar to those in the patients with SGS. When inflammation is accompanied by mucosal ulceration, hemoptysis may occur (29).

GPA affecting the airways occasionally leads to tracheo- or bronchomalacia (31). This situation may occur in cases of cartilage involvement or by stenosis. Tracheo- and/or bronchomalacia predispose the trachea or bronchi to dynamic collapse during expiration or inspiration. Patients may present with nonspecific symptoms like dyspnea, cough, or hemoptysis. In addition, they may develop recurrent infections and bronchiectasis, due to poor clearance of secretions (36).

Bronchiectasis is more prevalent in AAV with MPO-ANCA positivity (30), and associated with distinct phenotypes (female, older age, common nerve involvement and renal disease) (30). In GPA, bronchiectasis in imaging studies is found in about 20% of patients, but significant clinical features of this condition are described occasionally (29).

#### 3.1.3 DAH

According to various studies, the frequency of DAH in GPA cases ranges from 8.8 to 36% of patients (20, 37); however, it is frequent in MPA (25–60%) (38), and rare in EGPA (0–4%) (39, 40). This condition implies pulmonary capillaritis, leading to fibrinoid necrosis of the capillary walls with the loss of the integrity of the alveolar-capillary membrane, and resulting erythrocyte extravasation into the alveolar space with impairment of the gas exchange (23). The combination of DAH and kidney failure defines the pulmonary–renal syndrome, which presents in up to 97% of cases of MPO-AAV (41). DAH is a potentially life-threatening manifestation but milder forms also occur, and the course may be subclinical and recurrent. Symptoms are non-specific and include dyspnea of various degrees, hypoxemia, anemia, and hemoptysis, with the latter occurring only in about 50% of patients (10, 23, 26). Thus, DAH should be suspected in patients with GPA who have alveolar filling defects on chest imaging, independently of the clinical symptoms. Results of the systematic review showed that patients with GPA and presenting with DAH were 49.55 ± 17.54 years on average and were predominantly male (59%). Low hemoglobin levels were a constant clinical feature, and almost all patients had anemia at presentation, with only 61.5% reporting hemoptysis on presentation. Renal involvement and the need for mechanical ventilation were present in two-thirds of the cases (42). Factors independently associated with the development of respiratory failure include the high degree of hypoxemia, a high percentage of neutrophils in the bronchoalveolar lavage fluid (BALF) cell count, and high C-reactive protein (CRP) levels (43). Whereas the need for dialysis, low oxygen saturation < 90% at admission, need of mechanical ventilation (44), and age > 65 years (42) were the factors found as associated with unfavorable prognosis and higher mortality.

#### 3.1.4 ILD

The ILD is more likely to affect MPA than GPA. In MPA, ILD has been reported in 2.7–45% of patients, while in GPA this manifestation is present in about 23% of cases (45, 46). Furthermore, prevalence of ANCA in individuals initially presenting with isolated ILD ranges between 4 and 36% for MPO-ANCA, and only 2–4% for PR3-ANCA (45). It is suggested that MPO-ANCA may play a direct role in the pathogenesis of ILD, while PR3-ANCA seems not to be associated with ILD (47). Nonetheless, the presence of PR3-ANCA in patients with idiopathic interstitial pneumonia may be associated with a poor prognosis, similar to those with idiopathic pulmonary fibrosis (IPF) (47). ILD may occur in any time; that is before, simultaneously or after diagnosis of AAV (45, 46). Symptoms are non-specific and include progressive dyspnea and nonproductive cough, and physical examination typically reveals crackles, while digital clubbing are rather rare (23, 45). In most cases ILD progresses to pulmonary fibrosis, especially in MPA, while in GPA, ILD tends to be less aggressive and better respond to treatment (48). In the recent study of 684 AAV cohort (470 with MPO-ANCA and 214 with PR3-ANCA positivity), 13% of patients had ILD which preceded the diagnosis of AAV by a mean of 2.2 years. AAV-ILD patients were older, more often MPO-ANCA + (93% vs. 65%), and had a lower baseline disease activity than those without ILD. Indeed, among patients with MPO-ANCA + AAV, 18% had AAV-ILD, while among patients with PR3-ANCA+ AAV, only 3% develop AAV-ILD (49). The majority of patients had fibrotic ILD, with usual interstitial pneumonia (UIP) was the most common ILD type (42%), followed by fibrotic organizing pneumonia (OP) (21%), non-fibrotic OP (15%), and fibrotic non-specific interstitial pneumonia NSIP (11%) (49). In another study of AAV-ILD patients (56 with MPA and 39 with GPA), NSIP was the most common detected ILD (61%), followed by UIP (48%), and OP (10.5%), with NSIP was mainly observed in patients with c-ANCA positivity, while UIP was mainly found in patients with p-ANCA (50). In this study, ILD preceded vasculitis diagnosis in 22.1% of cases and mainly in p-ANCA positive patients (85.7%), while among those with c-ANCA, only one patient developed ILD before vasculitis (in 44.2% of cases the diagnoses were concomitant, and in 33.7% ILD followed the diagnosis of AAV). These results suggest the need for a regular assessment also for ILD in patients with AAV, particularly in those with GPA; on the other hand, patients with ILD and p-ANCA positivity (often initially classified as IPF) should be regularly assessed for the development of vasculitis (50).

Regarding the prognosis, many studies demonstrated that patients with MPA and ILD, but not with GPA, have worse prognosis compared to those with alone AAV, without ILD (51, 52). In one of the recent study, fibrotic AAV-ILD was associated with a 58% higher risk of death compared with AAV patients without ILD (49). In another study, patients with UIP patterns had an approximately 5-fold risk of death compared to those with NSIP (53). Factors identified as associated with mortality include chronic respiratory insufficiency, induction therapy with CS alone, and initial weight loss (54). In another study, an age > 65 years at AAV diagnosis, DAH at the time of AAV diagnosis, and an UIP pattern (compared to NSIP) were associated with shorter survival in AAV patients and ILD that those with alone AAV, without this manifestation (51).

### 3.2 Extrapulmonary involvement

**Figure 2:**
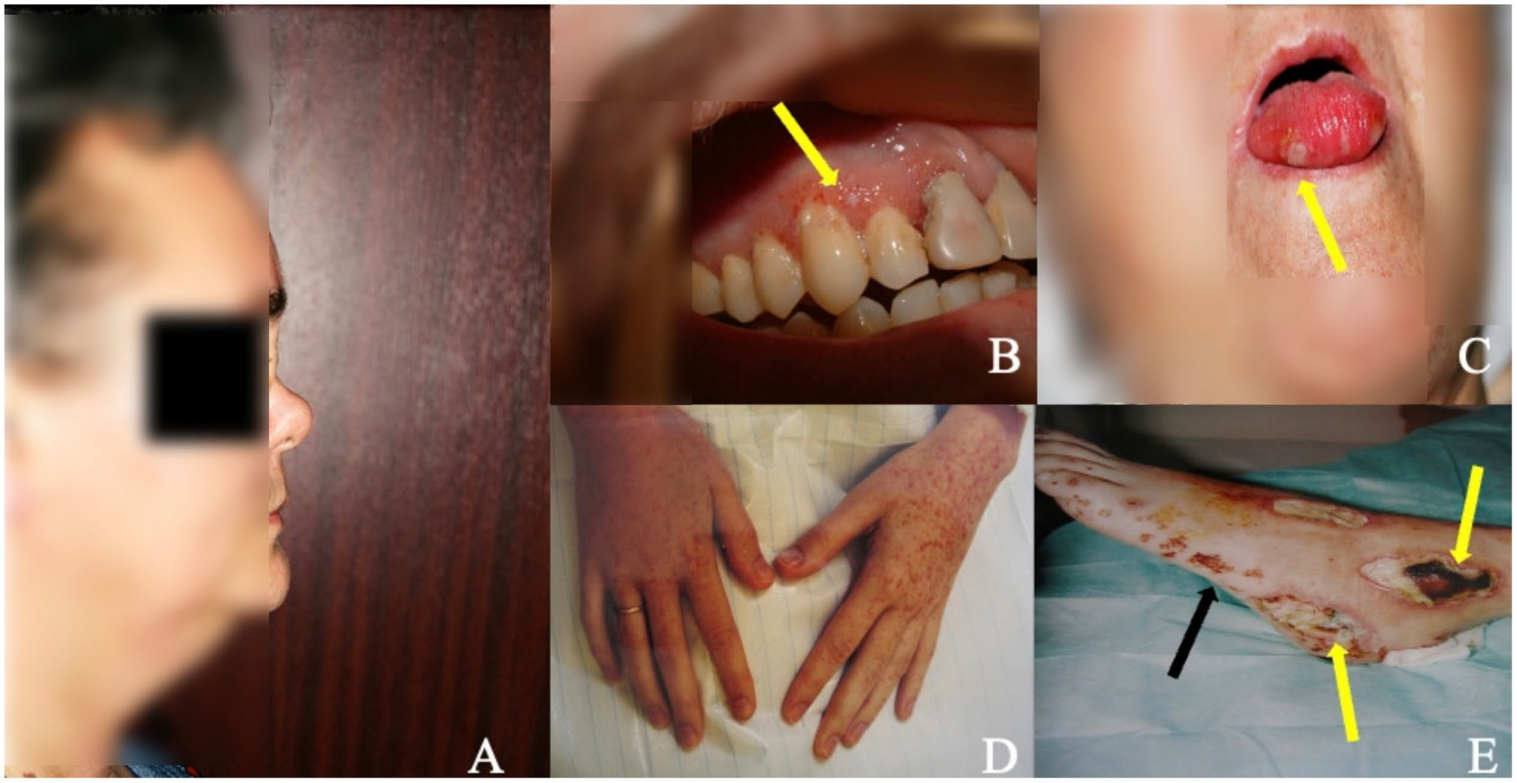
ENT involvement and skin/mucosal lesions in patients with GPA. **(A)** Saddle nose deformity (lateral view) in patient with long-term GPA and ENT involvement as a result of septal cartilage destruction with subsequent nasal collapse caused by granulomatous inflammation. **(B,C)** Mucosal lesions in the oral cavity in two patients with GPA manifesting in one as gingival hyperplasia with petechiae and small foci of necrosis **(B)** (arrow) and in the other as small necrotic lesions on the tongue **(C)** (arrow). **(D,E)** Purpuric lesions on the skin of hands **(D)** and of the left foot **(E)** (black arrow) accompanied by ulcerations and necrosis **(E)** (yellow arrows) in patient with generalised GPA.

ENT signs are present in 80–100% of cases (18, 20). The most prominent manifestations include nasal crust formation (75%), followed by excessive nose-blowing (70%), nasal obstruction (65%), epistaxis (59%), septal perforation, and saddle-nose deformity (Figure 2A) (27), the latter presenting in 25% of patients as a result of septal cartilage destruction with subsequent nasal collapse, caused by granulomatous inflammation (27). Ear involvement is almost always secondary to nasal involvement and leads to hearing loss, which may be the first GPA symptom. Involvement of the oral cavity is present in 6–13% of patients (55) (Figures 2B,C), with strawberry” gingival hyperplasia is the most common (61.5%) and most characteristics sign of GPA (55). Renal involvement occurs in 70–85% of patients during the course of the disease, while renal insufficiency occurs in 11–17% of patients at presentation (10) and 24.3% of patients experience worsening of glomerular filtration rate (GFR) over time (20). The clinical presentation is heterogeneous, from the indolent course progressing over months to years, to the rapidly progressive glomerulonephritis (GN) (20, 56). The frequency of cardiac involvement ranges from 1 to 61%, dependent on the study and diagnostic technique used (20, 22, 57–59); however, clinically overt cardiac involvement is reported in 3.3% of patients, with pericarditis and myopericarditis are the most common cardiac manifestation (58). Nevertheless, significant proportions of patients are asymptomatic or have few clinical symptoms, which may be easily overlooked (59, 60). Peripheral nervous system (PNS) involvement has been reported in 11–44% of GPA cases (61). The classic presentation is pain of acute onset, weakness, and sensory loss in the distribution of a named nerve (mononeuritis), followed by involvement of additional nerves in a stepwise fashion over weeks to months (multifocal neuropathy or mononeuritis multiplex) (62). Central nervous system (CNS) involvement is rarely seen in GPA (3–11.7%) (20, 22) and the most common form is hypertrophic pachymeningitis (HP) (10) typically manifesting as headaches and cranial neuropathies (10, 63). The autonomic nervous system (ANS) may also be involved (64). Clinical presentations of skin involvement encompass a wide range of symptoms (Figures 2D,E), however, pyoderma gangrenosum and palpebral xanthoma were identified as specific to GPA (65). Ophtalmological signs occur in 50–60% of patients (66), with orbital lesions (manifesting as pseudotumor or granulomatous inflammation) develop in 5–30% (67) and are considered the second most prevalent ophthalmic manifestation after conjunctivitis/episcleritis (67). Finally, gastrointestinal involvement has been reported in 1–26.5% of cases (20–22, 68, 69), with the most frequently symptoms include abdominal pain and bloody diarrhea (20, 21, 70).

## 4 Diagnosis, classification, and phenotypes

### 4.1 Diagnostic and classification criteria

To date, there are no validated diagnostic criteria for GPA, and there is no single test confirming the diagnosis. The Chapel Hill Consensus Conference (CHCC) provided a nomenclature classification and not a diagnostic classification (2, 71). However, in 2007 the British Society for Rheumatology (BSR) together with the British Health Professionals in Rheumatology (BHPR), proposed criteria which must be fulfilled to diagnose of AAV (72). In fact, current diagnosis of GPA is mostly based on clinical features, imaging findings, and supported, whenever possible, by the presence of ANCA and histological examination (73). Although many of the signs and symptoms discussed above can be seen in other diseases, multiorgan involvement is a key diagnostic clue, and the index of suspicion should be high if at least two of these symptoms are present (18).

Unlike the diagnostic criteria, the classification criteria have been developed for the purposes of clinical trials, to ensure that homogeneous populations are selected for inclusion. They are not appropriate for use in establishing a diagnosis of vasculitis, but to differentiate cases of GPA from other types of vasculitis; therefore, they should only be applied when a diagnosis of small or medium vessel vasculitis has been made, and other vasculitis mimics conditions have been excluded (74–76). The review of definition, diagnostic and classification criteria of GPA is presented in Table 1.

**Table 1:** GPA – definition, diagnosis, and classification.

Definition	Diagnosis	Classification		
CHCC 2012	BSR and BHPR 2007	EMA 2007	ACR 1990	ACR 2022
Necrotizing granulomatous inflammation usually involving the upper and lower respiratory tract, and necrotizing vasculitis affecting predominantly small to medium vessels; necrotizing GI is common Ocular vasculitis and pulmonary capillaritis with haemorrhage are frequent Granulomatous and non-granulomatous extravascular inflammation are common Limited expression also occur, especially confined to the upper or lower respiratory tract, or the eye	1. Symptoms and signs characteristic of systemic vasculitis 2. At least one of the following: a.histological evidence of vasculitis and/or granuloma formation b. positive serology for ANCA (either cANCA/PR3 or pANCA/MPO) c. specific indirect evidence of vasculitis 3. No other diagnosis to account for symptoms or signs All criteria must be fulfilled	1. Fulfilled 1990 ACR for WG or 2. Histology compatible with CHCC for WG or 3. Histology compatible with CHCC for MPA but WG surrogate markers* are present or 4. There is lack histology, but WG surrogate markers* and ANCA positivity are present * Fixed pulmonary infiltrates, nodules or cavitations present for >month, bronchial stenosis, bloody nasal discharge and crusting for >1 month or nasal ulceration, chronic sinusitis, otitis media or mastoiditis for>3 months, retro-orbital mass or inflammation,SGS Only one surrogate marker is necessary to support of diagnosis of GPA Surrogate markers for renal vasculitis: haematuria (red cell casts or > 10% dysmorphic erythrocytes) or haematuria (2+) and proteinuria (2+)	1. Nasal or oral inflammation (oral ulcers, purulent or bloody nasal discharge) 2. Abnormal chest X-Ray (nodules, fixed infiltrates, cavities) 3. Urinary sediment (microhaematuria >5 RBC) or red cell casts in urine 4. Granulomatous inflammation on biopsy granulomatous inflammation within the wall of an artery or in the perivascular or extravascular area At least 2 of 4 criteria are needed for classification of GPA	1.ENT symptoms: bloody discharge, ulcers, crusting, congestion, blockage, septal defect/perforation (+3 points) 2.Cartilaginous involvement: inflammation of ear or nose cartilage, hoarse voice, stridor, endobronchial involvement, saddle nose (+2 points) 3.Conductive or sensorineural hearing loss (+1 point) 4.Positive test for ANCA (cANCA or anti-PR3) (+5 points) 5.Pulmonary nodules, mass, cavitation (+2 points) 6.Granuloma, extravascular granulomatous inflammation, or giant cells on biopsy (+2 points) 7.Sinusitis or mastoiditis (+1point) 8.Pauci-immune GN in biopsy (+1 point) 9.Positive test for pANCA or anti-MPO (−1 point) 10.Blood eosinophilia ≥1×10^9^/liter (− 4 points) A score ≥ 5 is needed for classification of GPA

### 4.2 Diagnostic tools and differential diagnoses

As in GPA each organ may be affected, therefore, it is essential to conduct a detailed interview of medical history and perform diagnostic tests assessing the organ lesions (Table 2). As mentioned above, there is no single test to confirm GPA, therefore, the diagnosis is based on the combination of serological tests, imaging studies, and biopsies with histological examinations; however, all results should be interpreted in the correlation with clinical picture. Additional tests include endoscopies, particularly a flexible fiberoptic bronchoscopy (FOB), and pulmonary function tests (PFTs), which both are important informative procedures in patients with respiratory manifestations, with FOB allows to take biopsy and exclude other causes, for example infection or malignancy. All these techniques are not only of diagnostic importance but can also be used to monitor organ damage.

**Table 2:** GPA - organ manifestations, differential diagnoses, and diagnostic techniques.

Organ involved/main features	Clinical symptoms	Differential diagnoses	Diagnostic techniques
Lungs/pleura			
Nodules and masses (with or without cavitation)Pulmonary infiltrates/opacities/alveolitis DAH ILD Pleuritis	Cough (often with the sputum), hemoptysis, dyspnea, chest pain, fever Dyspnea until respiratory failure, hemoptysis (in part of cases), anemia Progressing dyspnea, dry cough, limited exercise tolerance Chest pain, dyspnoea	Acute or chronic bacterial infections (e.g., TB or other *Mycobacteria*), fungal infection, as aspergillosis or cryptococcosis (others such as histoplasmosis, coccidiomycosis, blastomycosis should be considered in endemic areas), Nocardia, aspiration pneumonia, malignancy, NSG, RA, COP, lymphomatoid granulomatosis, BALT lymphoma, pulmonary infarct Primary (anti-GBM disease, MPA, cryoglobulinaemic vasculitis), and secondary vasculitides (APS, SLE, RA, MCTD, drug-induced), other non-immune mediated causes (infectious, CHF, coagulation disorders, acute respiratory failure syndrome, inhalation of toxic substances, hemosiderosis) CTD, drug-induced, smoking-related, chronic HP, IgG4-related disease, idiopathic Infection, pleural effusion secondary to heart or renal failure	X-ray, CT, FOB with cytological and microbiological examinations, PFTs, lung biopsy X-ray, CT, FOB and BALF (determination of hemosiderin-laden macrophages), basic laboratory tests (including Cr, blood count), immunological examinations (e.g., ANA, anti-GBM and anti-phospholipids antibodies), cultures, ECHO, BNP, detailed medical interview Detailed medical interview, occupation, environmental exposure, drugs taking, immunological examinations (e.g., ANA, serum precipitins, anti-IgG-4 antibodies), PFT, FOB, BALF, lung biopsy USG, thoracocentesis with fluid examination and cultures, pleural biopsy
Trachea and bronchi			
SGS Mucosal abnormalities, masses and polyps, TBISs, trachea- or bronchomalacia, bronchiectasis	Dyspnea, stridor, hoarseness, cough, hemoptysis, recurrent infections	Polychondritis, inflammatory bowel disease, sarcoidosis, infection (especially fungal), asthma, allergy, neoplasm, amyloidosis, postintubation stenosis	CT, FOB, PFT, cultures, biopsy
ENT			
Chronic rhinosinusitis, often purulent and with bone destruction, nasal septum perforation Otitis media unilateral or bilateral, with the obstruction of the eustachian tube, which may be complicated by facial nerve palsy and mastoiditis	Nasal obstruction, sinus pain, nose deformity (collapse of the nasal bridge) Gradual or fluctuating hearing loss conductive (more common), sensorineural or mixed, otorrhea, earache	Chronic infection (especially TB and fungal), sarcoidosis, cocaine abuse, lymphoma	Complete ENT assessment (with nasal endoscopy), sinus CT, or MR (of the ear), laryngoscopy, nasal swabs cultures, audiogram, biopsy
Kidney			
GN (usually pauci-immune necrotizing, often crescentic) Interstitial nephritis	Microscopic and dysmorphic hematuria, sub-nephrotic proteinuria, with or without rapid serum Cr increase, hypertension, with or without azotemia Sediment alterations with or without Cr increase (acute or subacute)	Urinary tract infection, urolithiasis, CKD, CTD (e.g., SLE), hypertensive or diabetic nephropathy, bladder or renal malignancy, drug-induced kidney failure, anti-GBM disease	Urinalysis, 24-h proteinuria collection, blood urea, Cr, eGFR, anti-GBM, urine culture, USG, kidney biopsy
Heart/pericardium			
Pericarditis (the most common) Cardiomyopathy/myocarditis, valvular disease, conduction disorder Artery disease (vasculitis) Rare: coronary artery aneurysms, coronary artery dissection, tumor of the heart, endocarditis	Chest pain, dyspnea Chest pain, dyspnoea, palpitations, fever	Infection, CTD, pericarditis secondary to malignancy (e.g., lung or breast cancer) or radiation Cardiomyopathy related to atherosclerosis or hypertension, infective myocarditis	ECG, ECHO, serum troponin and BNP, 24-h ECG, cardiac MR (optionally FDG-PET/CT), rare cardiac biopsy CT or MR angiography, TEE, blood cultures, rare conventional angiography
Digestive tract			
Any organ may be affected Intestine, stomach Liver Spleen Pancreas Gallbladder	Abdominal pain and bloody diarrhea, nausea, vomiting, hematochezia or melena, dysphagia, perforation, may be subclinical course with positive occult blood in the feces Elevated liver enzymes, organ enlargement, rare vasculitis on the portal spaces and centrolobular territories Segmental infarction, rather asymptomatic Recurrent pancreatitis, pseudotumoral masses Cholecystitis, infarction	Infection, inflammatory bowel disease (e.g., CD, UC), peptic ulcer disease, malignancy, diverticulitis, neoplasm Pancreatitis of the other cause (alcohol, drugs, cholelithiasis), malignancy Cholelithiasis, infection, dietary mistake, other causes of acute abdominal pain (gastritis, appendicitis, biliary colic, peptic ulcer disease), neoplasm	Fecal occult blood tests (3x), liver enzymes (cholestatic and hepatocellular pattern), lipase, amylase, abdominal USG, CT/MR, endoscopy, biopsy Screening for HBV and HCV infection is recommended at the beginning of GPA diagnosis in all patients
Nervous system			
**Peripheral**			
Distal symmetrical sensory neuropathy, mononeuritis multiplex, peroneal, tibial, ulnar, and median nerves are commonly involved Cranial neuropathies (II-VIII)	Pain, burning sensation, numbness, limb weakness, foot or wrist drop and other sensory and/or motor deficits Often secondary to the external compression from ENT and/or orbit mass	Paraneoplastic, AIDS, DM, chronic liver disease, infection (Guillain-Barre syndrome), vit B6 or B12 deficiency, drugs, other type of vasculitis Tumor, stroke	Clinical evaluation, EMG, nerve biopsy, laboratory and serological tests MR
**Central**			
Brain parenchyma (symptoms related to vasculitis) Posterior reversible encephalopathy syndrome (PRES) (rare) Isolated parenchymal mass lesions (very rare) Cognitive impairment	Ischemic or hemorrhagic events Headache, seizures, altered mental status and visual loss Variable symptoms due to CNS compression, seizures are the most common Mostly subclinical or mild, affecting mainly abstract reasoning, attention and non-verbal memory	Vascular atherosclerotic disease, acute disseminated encephalomyelitis Primary and secondary degenerative diseases, age-related dementia, brain tumor, infection (AIDS) Primary (i.e., glioma) or secondary (metastases) brain tumors, infection (e.g., in the course of AIDS) Degenerative diseases (e.g., Alzheimer disease), age-related – dementia, infection	MR/MR angiography, cerebrospinal fluid examination and cultures, biopsy (rare)
**Meninges**			
Hypertrophic pachymeningitis of the brain Spinal pachymeningitis	Headache, dizziness, seizures, cranial neuropathy, e.g., paresis of the ocular motor nerves Paraplegia, truncal sensory abnormality, neck and back pain	Infection, neoplasms (e.g., lymphomas), sarcoidosis	
Skin/mucosa			
**Skin**			
Purpura, ulcers, nodules, pyoderma, gangrenosum - like lesions, other (e.g., livedo reticularis) **Mucous membranes** Strawberry gingivitis, oral or nasal ulcers, genital ulceration	Pain, necrosis Pain, bleeding, nasal crusting, purulent or blooding nasal discharge	Infection, allergic vasculitis, cutaneous vasculitis in CTD, Henoch – Schonlein purpura, HCV - induced vasculitis, cryoglobulinemic vasculitis, vasculitis associated with vasculopathic disorders (e.g., factor V Leiden mutation), immunodeficiency (e.g., AIDS), classic pyoderma gangrenosum, lymphoproliferative disorders, ulcerative colitis, pseudovasculitis	Clinical and serological evaluation, biopsy
Ocular			
**Orbit**			
Orbital or retro-orbital mass, usually unilateral, often coexists with sinus disease with bone destruction	Pain, proptosis, diplopia, eyelid swelling, periorbital cellulitis, ocular movement impairment, visual loss	Infection, lymphoma, sarcoidosis, cellulitis, cavernous hemangioma, venolymphatic malformation	MR, optic tomography, fluoroangiography, slit lamp examination, USG (in some cases), biopsy (rare)
**Eye** Conjunctivitis, scleritis, episcleritis, keratitis, retinal involvement	Red eye, tearing, corneal ulceration, blurred vision	Infection, CTD, sarcoidosis, IgG4-related disease, inflammatory bowel disease	
General/ Musculoskeletal			
	Fever, weakness, weight loss, malaise, myalgia, arthralgia, arthritis usually not-deformative	Infection (including TB), malignancy, CTD, polymyalgia rheumatica, hormonal disorders (e.g., hyperthyroidism)	Clinical evaluation, laboratory and immunological tests, hormone examinations, articular USG, cancer screening
Other rare involvements			
Urogenital			
Prostatitis, renal pseudotumor, orchitis, epididymitis, ureteral stenosis, penis ulceration	Pain, swelling, fever, difficulty urinating	Infection, neoplasm, benign prostatic hyperplasia	CT or MR, cystoscopy, biopsy
Endocrine			
Salivary glands Lacrimal glands Pituitary Thyroid Adrenal glands	Swelling, pain, necrosis, dry mouth Pain, tearing, reddening Cranial diabetes insipidus, hypogonadism, secondary hypothyroidism, hyperprolactinemia, growth hormone deficiency, polyuria, polydipsia, decreased libido, muscular atrophy, asthenia, amenorrhea, compressive symptoms (headache, vomiting, visual field loss) Hypothyroidism Adrenal insufficiency	Infection, Sjogren syndrome Infection, allergy Adenoma, lymphoma, metastasis, radiation, LCH, post-traumatic damage, stroke, sarcoidosis Hashimoto disease, cancer, subacute thyroiditis, drug-induced CS treatment, infection (TB), autoimmune adrenal inflammation, stroke of the hypothalamic–pituitary region, neoplasm	Clinical evaluation, USG, MR, biopsy Clinical evaluation MR, hormone examinations USG, hormone and serology examinations, scintigraphy, biopsy USG, MR, hormone examinations, biopsy (rare)
Breast			
Breast	More common in women, pain, ulceration	Breast cancer	Biopsy is decisive
Vertebral			
Paravertebral masses, usually not associated with bone erosion or compression of vessels	Often asymptomatic, sometimes back pain	Cancer, lymphoma	MR
Large and medium-sized vessels			
Thoracic and abdominal aorta (periaortitis the most common), carotid and subclavian artery, pulmonary arteries, renal arteries	Stenosis, occlusion, aneurysms, periaortic mass, thoracic or abdominal pain, fainting, stealing syndrome, low cardiac output syndrome, decrease of blood pressure, may be asymptomatic	Isolated large vessel vasculitis, atherosclerosis, thrombosis, congenital	CT/MR angiography, optionally FDG-PET/CT, conventional angiography (rare)

GPA, granulomatosis with polyangiitis; CT, computed tomography; FOB, fiberoptic flexible bronchoscopy; PFTs, pulmonary function tests; RA, rheumatoid arthritis; COP; cryptogenic organizing pneumonia; BALT, bronchus-associated lymphoid tissue; DAH, diffuse alveolar hemorrhage; anti-GBM, anti-glomerular basement membrane antibodies; MPA, microscopic polyangiitis; BALF, bronchoalveolar lavage fluid; APS, antiphospholipid syndrome; SLE, systemic lupus erythematosus; CTD, connective tissue disease; MCTD, mixed connective tissue disease; HP, hypersensitive pneumonia; ANA, anti-nuclear antibodies; Cr, creatinine; ECG, electrocardiography; ECHO, echocardiography; BNP, brain natriuretic peptide; USG, ultrasonography; ENT, ear, nose, throat; MR, magnetic resonance; FDG-PET/CT, F-18 fluorodeoxyglucose - positron emission tomography; TEE, transesophageal echocardiography; HBV, hepatitis B virus; HCV, hepatitis C virus; AIDS, acquired immune deficiency syndrome; DM, diabetes mellitus, CNS, central nervous system, TB, tuberculosis; AIH, autoimmune hepatitis; PSC, primary sclerosing cholangitis; PBC, primary biliary cholangitis; CD, Crohn’s disease; UC, ulcerative colitis.

Of note, the exclusion of other causes is an important component of GPA diagnosis; however, when the diagnosis remains uncertain, observation over time, repeat investigation, and a therapeutic trial may improve the probability of the GPA diagnosis or identify an alternative disease (77).

#### 4.2.1 Laboratory testing

##### 4.2.1.1 Routine laboratory tests

In patients with suspected GPA, the following routine laboratory tests should be performed: complete blood count with differential and blood smear, renal function (creatinine with eGFR and urea), electrolytes, liver enzyme, coagulation tests (with D-dimer), erythrocyte sedimentation rate (ESR) and CRP, and complete urinalysis with urinary sediment and 24-h proteinuria collection (73). As cardiac involvement may be often asymptomatic, the determination of basic cardiac markers such as troponin T and brain natriuretic peptide (BNP) should be considered. Results of routine tests are usually non-specific, however, in GPA, as in other inflammatory diseases, patients usually have leukocytosis with increased neutrophilia, normocytic normochromic anemia (as an expression of inflammation), thrombocytosis and increased CRP or ESR. The latter can be normal even in active GPA, if limited to a single organ disease (10).

Other laboratory studies, which should be performed include immunoglobulins concentrations, complement (C3, C4), serum protein electrophoresis, QuantiFERON test, viral serologies, such human immunodeficiency virus (HIV), and hepatotropic viruses (hepatitis B virus; HBV and hepatitis C virus; HCV), and ANCA serology with target antigens (78).

##### 4.2.1.2 ANCA testing

ANCA is an important diagnostic marker in patients with AAV (1, 79) and should be performed in every patient with clinical features suggesting AAV (80). There are reports indicating that these antibodies may be present years before AAV symptoms to support the important role of ANCA in AAV pathogenesis (81). Alternatively, ANCA is not a specific marker for AAV because it can be detected in other conditions (82). In addition, the absence of ANCA does not exclude AAV, when it is supported by clinical symptoms and histological examination, after excluding other causes (80). The recent meta-analysis demonstrated that PR3-ANCA has greater sensitivity than MPO-ANCA for GPA (74% vs. 11%), while MPO-ANCA has greater sensitivity for MPA (73% vs. 7%), with consistently high specificities of both types of ANCA (mean 97%; range 93–99%) (83). According to current international consensus on ANCA testing, the use of antigen-specific assays for PR3- and MPO-ANCA is recommended as the first line of testing over indirect immunofluorescence (IIF) method in patients suspected AAV (80). Some studies demonstrated, that a higher ANCA titer and multiple affected organ systems may help to discriminate between AAV and other systemic diseases in anti-PR3 and anti-MPO positive patients. Using four different immunoassays for the ANCA test, ≥4 times the upper limit was a reasonable cut-off point to discriminate between AAV and alternative diagnoses (84). However, ANCA is not a substitute for the biopsy and has an important role in suspected cases when histological confirmation cannot be obtained (33).

Although ANCA is a valuable diagnostic tool of GPA, in about 8.5% of patients ANCA serology results are negative (20). In the absence of ANCA, the diagnosis may be difficult to assess, and new reliable biomarkers for diagnosis of AAV, particularly in seronegative cases, are being sought. Anti-pentraxins (anti-PTX3) antibodies may become a promising candidate as a novel diagnostic biomarker of AAV, including patients with negative serology for MPO- and PR3-ANCA. High levels of PTX3 have been found in active AAV, large vessel vasculitis, and connective tissue diseases (CTDs), as in systemic lupus erythematosus (SLE) (85). Next, in the study focusing on AAV only, plasma and urinary PTX3 correlated with disease activity, particularly in patients with renal involvement (86). Finally, anti-PTX3 antibodies have been detected in almost 40% of AAV patients, with half of the patients with undetectable MPO- and PR3-ANCA, were positive for anti-PTX3 (87).

#### 4.2.2 Imaging techniques

Further evaluation should be made of the affected organs, using imaging as an integral part of the diagnostic evaluation in patients with GPA to detect organ involvement and to identify potential biopsy sites (73). Furthermore, chest imaging should be performed in any patient in whom GPA is suspected, since up to one-third of patients may be asymptomatic yet have pulmonary radiographic findings (88). Imaging abnormalities in GPA are non-specific, and overlapping patterns are common, moreover, lesions may evolve with the stage of the disease (active or chronic persistent lesions) and treatment (89). Knowledge of the clinical background of the patient, experience, and close cooperation between clinician and radiologist are necessary for the appropriate assessment of imaging lesions and further management.

##### 4.2.2.1 Conventional X-ray

Chest X-ray is the preliminary screening examination for pulmonary involvement in GPA and should be performed in every patient suspected of GPA regardless of whether there are respiratory symptoms or not. However, chest X-ray has limited sensitivity, and some lesions may not be visible as well as a precise determination of the nature of lesions or their precise location may not be possible. Abdominal X-ray may be used as screening in cases of acute abdominal pain in patients suspected of gastrointestinal involvement. With this method pneumoperitoneum can be identified, and using oral contrast medium, increased wall thickness could be visualized (70). In turn, sinus X-ray has low diagnostic value, however, acute sinusitis with the fluid level may be visible.

##### 4.2.2.2 CT imaging

###### 4.2.2.2.1 Conventional CT and CT angiography

CT is more sensitive than conventional radiographs and is the technique of choice for the characterization of manifestations in GPA, especially in patients with respiratory symptoms, as it presents high diagnostic performance in paranasal cavities and ear pathology (showing both features of inflammation as well as bone destruction and intracranial extension of nasal lesions), and in the assessment of the thorax, especially the lung, tracheobronchial tree, mediastinum, and vascular structures (90). In addition, in some cases, chest CT may help to distinguish GPA manifestations from infection and other comorbidities (73). This technique allows for a precise assessment of the morphology and distribution of lung lesions and is helpful in selecting the biopsy site. The most common manifestation related to GPA include nodules and masses with or without cavitation, and usually at random distribution; followed by ground-glass opacities (diffuse or local, related to alveolitis or DAH), parenchymal consolidations, and tracheal or bronchial abnormalities as thickening wall and stenosis; rarer findings include pleural changes and ILD (24, 25, 49, 50, 53); the latter corresponding primarily to NSIP, UIP or OP patterns (49, 91) (Table 3). In up to 15% of patients, enlarged mediastinal lymph nodes may be found which are usually seen in association with lung abnormalities (92). In some cases, the mosaic attenuation or the “tree – in – bud” pattern is present which may indicate pulmonary arterioles involvement secondary to GPA (27). As conventional CT is the preferred method for large airway disease detection, the competitive technique is the dynamic expiratory chest CT which has been revealed as a potential screening test to evaluate for SGS and tracheobronchomalacia, where it has been demonstrated to better evaluate degree of collapse than other imaging (32). Whereas, CT angiography is useful in the involvement of large vessels, rarely seen in GPA, as aneurysms and aortitis manifesting as diffuse thickening of the arterial wall with edema and delayed enhancement (93).

**Table 3:** Features of main CT-patterns of GPA-related ILD.

NSIP pattern	UIP pattern	OP pattern
1. Bilateral ground-glass areas predominantly located in the middle and lower lung with or without a. Reticular opacities (which can be superimposed on a ground-glass pattern) b. Traction bronchiectases 2. Honeycombing rare 3. Subpleural areas usually not involved	1. Basal and subpleural predominance 2. Reticular pattern, with associated traction bronchiectasis 3. Honeycombing appearance 4. Absence of features* listed as inconsistent with UIP pattern *Upper or mid-lung predominance, peribronchiovascular predominance, extensive ground-glass abnormality, profuse micronodules, discrete cysts, diffuse mosaic attenuation/air-trapping, parenchymal consolidations	1. Peripheral or peribronchial patchy consolidations, often with air bronchograms and mild cylindrical bronchial dilatation 2. Ground-glass opacities with tendency to migration, change of location and size 3. Rarely mass or nodule that may cavitate or reproduce the typical appearance of an “atoll sign” (the presence of a ring consolidation surrounding normal lung or ground-glass opacification), considered as relatively specific for OP

CT, computed tomography; GPA, granulomatosis with polyangiitis; ILD, interstitial lung disease; NSIP, non-specific interstitial pneumonia; UIP, usual interstitial pneumonia; OP, organizing pneumonia.

In patients suspected of gastrointestinal involvement, abdominal CT might be a useful diagnostic tool and should be considered; however, the signs are non-specific and have to be correlated with clinical symptoms and patient medical history (70). In GPA, CT findings of bowel involvement include bowel dilatation and wall thickening, abnormal bowel wall enhancement, mesenteric vessels in a comb-like configuration, ascites, lymphadenopathy (27) or mesenteric fat haziness stemming from inflammation of the mesenteric vessels (94). Abdominal CT can also visualize splenic infarction because of diffuse arteritis resulting in occlusion of distal parenchymal splenic arteries. In renal involvement, this technique is usually negative, except when it presents as a nodule or renal mass (90).

###### 4.2.2.2.2 F-18 fluorodeoxyglucose (FDG) - positron emission tomography (Pet)/CT

There are reports about a high sensitivity of FDG-PET/CT in AAV, especially in active GPA. In one of the cohorts of mixed AAV, FDG-PET/CT at baseline was positive in 100% of GPA patients, and sinonasal, lung, cardio-vascular and kidney involvements were all accurately identified by FDG-PET/CT (95). In another study of GPA, lesions of respiratory tract and lung were more clearly detected by FDG-PET/CT than by conventional CT scan alone, with high sinus mucosal, cartilaginous and bone uptake in early stages, even with normal CT (96). The FDG-PET/CT has also shown high positivity in cardiac involvement with great concordant with cardiac magnetic resonance (MR) lesions (95). Furthermore, the correlation between FDG uptake intensity and GPA activity has been also reported demonstrating that this method might be useful for the control treatment and follow-up in AAV patients (95). However, although FDG-PET/CT has many advantages, it has several imperfections. Despite this, it accurately identifies organ localizations in GPA, it does not bring additional benefit to the usual organ screening. In addition, its sensitivity of skin, eye, and nervous system involvement is significantly low (95). Finally, findings are non-specific, and the FDG uptake intensity does not distinguish GPA from other entities, such malignancy, infection, or other granulomatous processes (96). For now, FDG-PET/CT should not be carried out routinely for the assessment of the spread of GPA, nor for its monitoring. On the contrary, it cannot be ruled out that in certain specific situations, this method will find a place in the future to distinguish active lesions from fibrous sequelae (e.g., orbital mass during GPA) (97).

###### 4.2.2.2.3 MR imaging and MR angiography

MR imaging is most useful in cardiac, encephalic, and ocular manifestations in GPA patients (90). Cardiac MR is a recommended imaging tool for diagnosis of suspected myocarditis and is most accurate in diagnosing cardiac lesions in vasculitis (98). In patients with clinically silent cardiac involvement, this technique is of great value in early diagnosis and follow-up (99, 100). This method provides essential information about the morphology and function of cardiac structures and has also a unique potential for myocardial tissue characterization, identifying inflammation and fibrosis, myocardial and vessel acuity and evaluating biventricular function (99). However, specific abnormalities associated with GPA remain undefined, and correlation with patient’s clinical background, laboratory and other findings is necessary. The late gadolinium enhancement (LGE) lesions are the most common cardiac MR findings in GPA patients as are detected in one-third of cases (60, 101). This pattern corresponds to myocardial fibrosis (99) and may be associated with cardiac damage (100, 101) and higher mortality (39, 59).

MR imaging combined with MR angiography is the first-choice method of central nervous assessment in GPA patients, including meningeal involvement, brain parenchyma lesions and cranial nerves involvement (27, 90), while MR angiography may visualize the vessels’ lumens, and stenoses, occlusions or aneurysms (102). Regarding to meningeal involvement, two MR patterns have been identified: diffusely abnormal meninges (unrelated to sinus or orbital disease), and focal dural thickening and enhancement adjacent to sinus or orbital disease (27). Whereas, typical findings of vasculitis in brain parenchyma include luminal stenosis alternating with dilatation (the “beading sign”), while secondary signs comprise structural damage of brain tissue as a result of the ongoing inflammation (multiple small, microvascular infarctions in different vascular territories and of different ages, visible as white matter lesions with patchy high T2 signal intensity) (102). Functional MR may be relevant as an additional diagnostic tool, as it can provide information on both – structure and potential damage of the brain as well as the function of the selected brain centers (64).

Orbital involvement is the next GPA manifestation where MR imaging has a high diagnostic value, demonstrating orbital lesions, involvement of adjacent structures, and the extent of tissue damage. This examination is complemented with CT, as CT offers the ability to depict sinus structure disorders and osseous invasion, while MR is helpful for identifying granulomas and delineating orbital mucosal changes (103).

The MR/MR angiography remains the preferred imaging modality in large vessels assessment, although, CT combined with CT angiography is an alternative technique to MR, and according to some authors, the preference of one method over another will depend on its availability, and factors such as contraindications to the iodine contrast, presence of artifacts or considerations regarding radiation (90). However, MR angiography of large vessel can provide functional data such as flow volume and velocity, which is a major advantage compared to CT angiography (104).

###### 4.2.2.2.4 Ultrasonography (USG)

In GPA, USG is reserved primarily for the assessment of extrapulmonary (e.g., kidney, liver) and thoracic involvement. Cardiac ultrasonography (echocardiography; ECHO) is recommended as the first-choice modality to screen cardiac involvement in AAV patients (73) as evaluating atrial and ventricular masses, valves lesions, left ventricular wall motor abnormalities and abnormalities of pericardium (105). Regarding the lung lesions, the efficacy of USG is well documented in many pulmonary conditions, such as pneumonia, pneumothorax, or pulmonary edema (106) while its efficacy in AAV-related lung lesions is not well established. However, in the recent study examining the utility of USG in AAV patients, it has been shown a high consistency of this method with CT findings (107). In turn, ultrasound examination of the pleural space is a highly sensitive and specific diagnostic tool in cases of pleural involvement and pleural effusion, allowing for the designation of a biopsy site, if needed and monitoring fluid dynamics (106).

#### 4.2.3 Endoscopy

FOB is indicated for GPA patients who have respiratory symptoms, abnormalities on pulmonary function testing, or abnormal findings on chest imaging (26). Samples obtained during FOB for microbiology and histology examinations are important parts of the differential diagnosis of lung nodules and masses. FOB is the gold standard for the diagnosis of airway involvement. It allows for direct visualization of the tracheobronchial tree, detecting mucosal lesions, SGS, bronchial stenoses and mass-like granulomatosis (108). It may detect also not advanced inflammatory lesions that have only subtle pulmonary consequences, but may explain some clinical symptoms, such as cough or localized wheezing (23). In addition, bronchoscopy allows to obtain a biopsy and precise mapping of inflammatory lesions before therapeutic interventions (23, 26, 108) as well as it is useful tool to treat and monitor tracheobronchial lesions.

FOB with BALF examination is the gold standard procedure to diagnose DAH (23, 26, 108) and might confirm the diagnosis despite the absence of clinical or biological arguments even in 16% of patients (108), with an increasingly hemorrhagic BALF after sequential sampling is specific for DAH and is the best diagnostic test (46). In addition, BALF examination is a valuable to rule out other etiologies for pulmonary infiltrates (infections in particular) (23, 26, 108). Classically, BALF confirms DAH with one of the following features: bloodier BALF return, Golde score > 20 (>100 for severe) or hemosiderin-laden macrophages >20%. However, DAH may have a subclinical course, with 5% defined as the upper limit of hemosiderin-laden macrophages (108). Although FOB is necessary for the unequivocal diagnosis of DAH, it could temporarily worsen the patient’s respiratory status. Therefore, it is essential to make sure before performing this procedure, that there are facilities to upgrade the level of care of patients, including the possibility of intubation and mechanical ventilation (26, 108).

The role of endoscopy of the diagnosis of gastrointestinal involvement in GPA patients is being debated because findings are not specific and it is common with normal macroscopic findings even in cases with severe involvement (70); however, it can be of differential diagnostic importance and should be considered in patients with gastrointestinal manifestations. The most frequent findings include erosions and ulcers with obstructed blood flow involving mainly gastroduodenal and colonic tract, however, esophagus may be also affected (109, 110). Other endoscopic features are nonulcerative uncharacteristic inflammations manifesting as red and swollen mucosa in affected parts of the digestive tract (70).

In patients with ENT manifestations, nasal endoscopy should also be considered. It usually reveals crusting, friable erythematous mucosa, and granulation. In turn, laryngoscopy is an alternative technique for SGS confirmation and grading in GPA patients (111).

**Figure 3:**
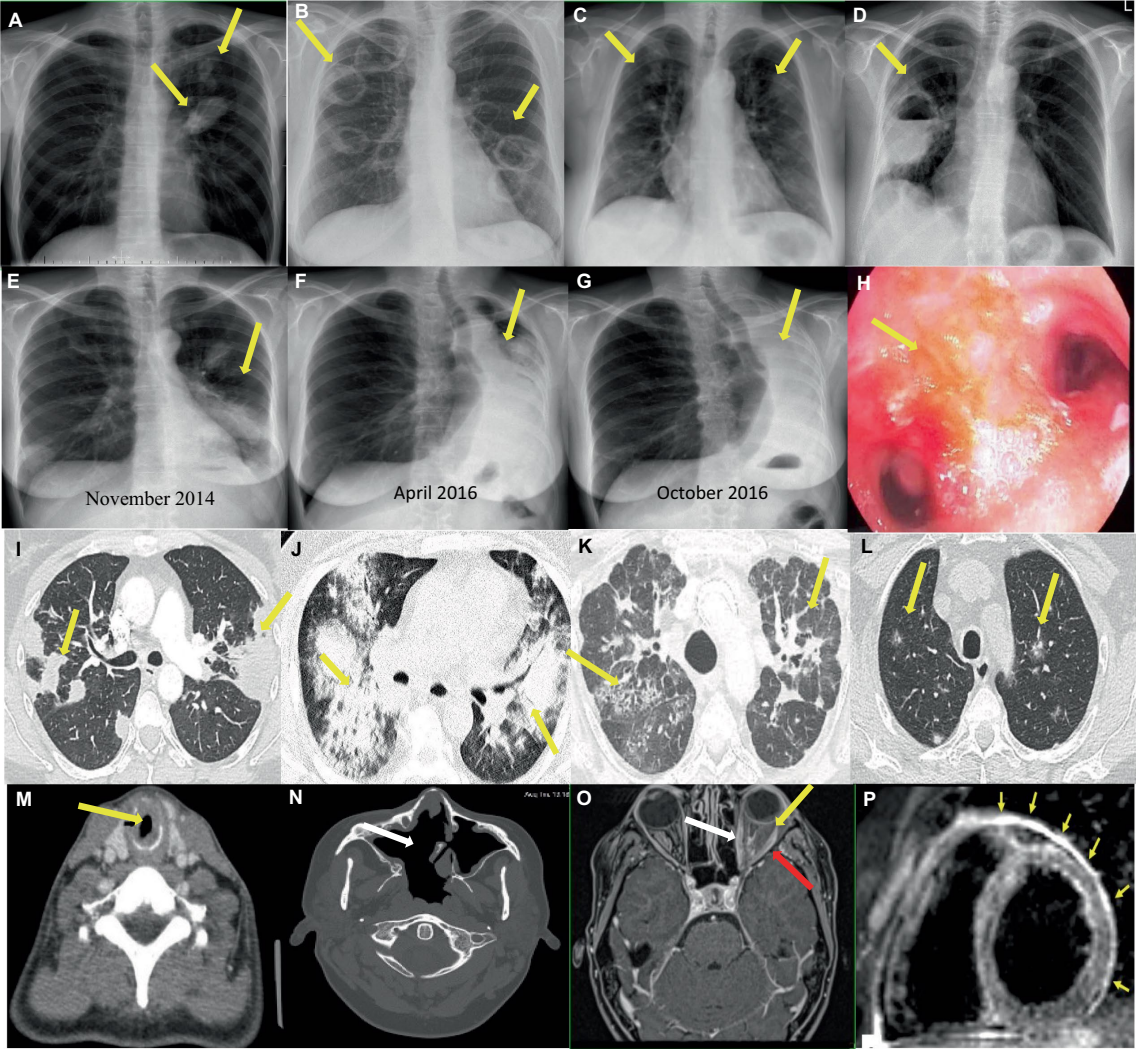
Imaging findings in patients with GPA. **(A–G)** Chest x-rays showing **(A)** Two nodular lesions (arrows) in the hilum and in the upper field of the left lung suspected initially for lung cancer. **(B)** Bilateral tumors with cavities (arrows) and **(C)** spread small lung nodules. **(D)** A thick-walled cavity with a fluid level in the right lung (arrow) suspected initially for lung abscess, and then for mycetoma. **(E–G)** Increasing obstruction of the left main bronchus **(E,F)**, up to its complete closure and the formation of atelectasis of the left lung (arrows) **(G)** in the patient with tracheobronchial involvement. **(H)** Endoscopic image of endobronchial lesions (arrow). **(I–L)** Chest CT scans showing **(I)** Bilateral parenchymal infiltrations and consolidations (arrows) corresponding to OP pattern (axial image, lung window). **(J)** Bilateral ground-glass opacities in patient with diagnosed DAH (axial image, lung window). **(K)** Features of ILD (NSIP pattern) (arrows), which appeared after many years of suffering from vasculitis (axial image, lung window). **(L)** Bilateral small and randomly distributed nodules with “ground-glass” component (arrows) corresponding to GPA in histological examination (axial image, lung window). **(M–N)** Head and neck CT scans showing **(M)** SGS in the course of GPA - tracheal narrowing and thickening of the tracheal wall (arrow) (axial image). **(N)** A large single sinus cavity (arrow) as a result of the destructive granulomatous process of the nasal septum (axial image). **(O–P)** Contrast-enhanced MR showing **(O)** orbital mass in the left orbit (yellow arrow) with extraocular muscle enlargement (red arrow) and optic nerve sheath enhancement (white arrow) (T1-weighted, axial image). **(P)** Myocardial edema and thickened pericardium with hyperintense signal (arrows) corresponding to cardiac and pericardial involvement in the patient with GPA (black blood T2-weighted STIR images in short axis plane).

Selected imaging findings in patients with GPA shows Figure 3.

#### 4.2.4 Biopsy and histology examination

Biopsy remains a gold standard of diagnosis GPA and is strongly recommended (73, 76). However, the biopsy may not be feasibly obtained in every patient and starting of treatment should not be delayed by waiting for histological examination (73, 78). The role of the biopsy may be particularly important in cases when ANCA is negative, and in limited form of the disease. Major histological features characterized GPA include necrosis, granulomatous inflammation, and vasculitis (112); however, it is rare for biopsy specimens to present with all three of them (113) and biopsies may show atypical or incomplete histological features, therefore correlation with clinical, serological, and radiological findings, and experience and close collaboration of pathologist and clinician are necessary to make the correct diagnosis (114). Of note, the diagnostic yield of the histological examinations may vary significantly depending on the biopsied site (78), and in many cases biopsies of affected organs must be repeated several times to confirm diagnosis (115, 116). Furthermore, the lack of typical histological features does not exclude GPA.

Regarding the respiratory tract, the sensitivity of biopsies is not satisfactory. Although the upper respiratory tract is easily accessible, biopsy of this localization shows non-specific inflammation even in 61% of patients (117), while only in 24% biopsy is diagnosed as typical of GPA, and diagnosis could be confirmed in 42% as typical of GPA from the results of multiple biopsy specimens (116). In turn, in patients with pulmonary lesions, in cases of transbronchial lung biopsies the diagnostic yield is about 12% (118). Whereas, in cases of visible endobronchial lesions, bronchoscopically obtained samples may support the diagnosis in about 50% of patients when other supportive features are present, while only in 25% are diagnostic by themselves (26). Surgical lung biopsy provides a greatest diagnostic value (117), but it is no longer used routinely due to the risk of serious complications; however, in patients with isolated pulmonary lesions that cannot be clearly attributed to GPA, thoracoscopic or open lung biopsies should be considered (73).

In contrast, to the respiratory tract, the diagnostic yield of a kidney biopsy in AAV patients can be as high as 91.5% (119). According to current guidelines, kidney biopsy should be performed where possible, however, in the context of positive MPO- or PR3-ANCA serology and a clinical picture compatible with AAV, an immediate biopsy may not be necessary and should not delay the initiation of treatment (73, 119). Biopsy specimens in GPA usually show a pauci-immune GN, with segmental necrotizing pattern (often crescentic) and extra-capillary proliferation are the most common findings (85.1 and 91.5% respectively) (120). The histopathological subtypes of kidney involvement do not guide treatment decisions (73), however, kidney biopsy provides prognostic information (121, 122), while repeated kidney biopsy may be useful to differentiate recurrent or refractory disease activity from damage or other alternative diagnoses (123).

Although the nerve biopsy remains the gold diagnostic standard, diagnosis of peripheral neuropathy is mainly based on clinical evaluation and can be confirmed by electrodiagnostic studies (124). In a large cohort of 955 AAV patients (DCVAS study), only 12% had nerve biopsies, of which 53% had definitive vasculitis (125). The most common nerves chosen for this procedure include the sural, superficial peroneal, and superficial radial nerves (126). The histological findings are characterized by the axonal degeneration of the nerve fibers and inflammation of the epineurial vessels accompanied by the destruction of vascular structures (126). Diagnostic yield of muscle biopsy is 51%, with factors predicting of diagnostic accuracy include ANCA type (MPO), sex (female) and higher neutrophil count (127).

Skin biopsy, while underutilized (performed in 22–44% of patients), is frequently found to be an effective test suitable for diagnosis of AAV (diagnostic in 68–94% of patients) (128). In the large study of 1,553 patients with mixed AAV, pathological analysis showed vasculitis and/or granulomatous infiltrates in 87.5% of GPA patients with skin manifestations, with vasculitis was more frequently observed in purpura and nodules, while granulomas were differently located and organized within vessels or the interstitium according to the type of lesions (129). Although these lesions are specific for GPA, they do not occur exclusively in GPA and can also be seen in other vasculitic disorders (65).

Cardiac biopsy is rarely performed to confirm cardiac involvement in GPA, as this manifestation is usually recognized based on the clinical background and non-invasive imaging modalities (ECG, ECHO, and cardiac MR) (73). In cases with known GPA, multiorgan manifestation and ANCA presence, the diagnosis is quite straightforward, and biopsy can usually be avoided. However, when symptoms are not attributable to GPA or cardiac disease is the only manifestation of vasculitis, cardiac biopsy may be necessary to confirm diagnosis (130). However, typical histological GPA features are rarely found (in less than in 2% of patients) (113). The low diagnostic accuracy is likely due to the rates of sampling errors. Additionally, the patchy cardiac lesions might complicate diagnosis (131).

Finally, the histopathological confirmation of gastrointestinal vasculitis is also difficult to obtain. Tissue samples collected during endoscopy usually reveal nonspecific inflammation or ulcers (69), while vasculitis can be detected histologically only in 8% of patients (109). Some authors speculate that this may be a result of biopsy taken too superficially during the endoscopy procedures, as the small and medium vessels, typically involved in GPA, are located deeper in the submucosa (69). Indeed, the results may be better for surgical removed specimens where signs of necrosis related to vasculitis may be found even in 90% of cases (109).

#### 4.2.5 Pulmonary function tests (PFTs)

PFTs are an integral part of the examination of the GPA patients, especially when respiratory symptoms or radiographic abnormalities of the lung or airway are present. The most common findings include airflow obstruction (40% of cases) (132) often in association with reduced diffusion lung capacity for carbon monoxide (DLCO), especially when accompanied by ILD (26). However, an alteration in the DLCO may be the first sign of DAH (26). In SGS, typically, the flow-volume curve shows a flattening in both respiratory and expiratory phase, consistent with an extrathoracic airway obstruction (28). It has been demonstrated the correlation between PFTs results and chest imaging findings in AAV patients. Patients with pulmonary fibrosis suffer more often from impaired DLCO whereas patients who show signs of lung consolidation, had a high risk for a restrictive pattern (133). Interestingly, it has been shown that abnormalities in PFTs in GPA patients are not always related to pulmonary involvement. In the study of 147 mixed AAV, for GPA, forced vital capacity expiratory (FVCex), residual volume (RV) and DLCO were statistically lowered compared to expected values of 100% predicted, and there was no significant difference between patients with or without pulmonary manifestations. In contrast, in patients with MPA, relevant impairments of FVCex, total lung capacity (TLC) and DLCO were observed compared to the standard population, and these changes were significantly stronger in MPA-patients with pulmonary involvement (133).

### 4.3 Differential diagnoses

As GPA has a multisystemic nature, the differential diagnosis is broad, and many conditions could mimic GPA, and should be ruled out before a final diagnosis can be established (Table 2). Differentiation is particularly difficult when the disease is confined to one organ. For example, isolated lung disease must be primarily differentiated with infection and malignancy, with the tests should include tuberculosis and fungal infections. Similarly, in the isolated sinus disease, infectious cause as the first should be excluded (88). On the contrary, infection can coexist with GPA and does not exclude active vasculitis. The appearance of the air-fluid levels in the lung cavities may indicate that they become infected, what should lead to a careful microbiological evaluation with subsequent targeted antibiotic therapy (26). Cavities may be also colonized by fungi - mostly *aspergillus fumigatus*, what may pose a particular difficult diagnostic challenge, because the radiographic features of fungal infections are often similar to those of GPA, and tests for fungal infections are often negative in patients without criteria for invasive aspergillosis (23). Next, pulmonary consolidations, reflecting the NGI, may be radiographically indistinguishable from pneumonia and organizing pneumonia (OP), with the latter representing the diagnostic challenge (26). Namely, the radiological pattern is very similar and practically indistinguishable from GPA limited to the lung and manifested by non-cavitated nodules or pulmonary consolidations. In addition, OP responds very well to CS treatment, similarly as GPA; and finally, OP pattern can occur in GPA (26).

Due to the different management, outcome, and prognosis, differentiating GPA from other AAV is also important. Despite GPA, MPA and EGPA belong to the same group of vasculitis, they have some unique features (Table 4) (88). However, polyangiitis overlap syndromes may occur, when the disease does not fit precisely into a single category of vasculitis classification and/or overlaps with more than one category (76). Particularly, differentiating between GPA and MPA can be demanding because of significant overlap in the signs and symptoms and in the ANCA serologies. In addition, patients initially presenting with only symptoms consistent with MPA, could later develop manifestations more compatible with GPA. A key distinguishing feature of these entities is the presence of granulomatous inflammation in GPA and its lacking in MPA (2).

**Table 4:** Differentiating GPA from other AAV.

GPA	MPA	EGPA
Typically ENT, lung and kidney involvement (ELK) ENT-necrotizing, destructive lesions Lung – nodules, masses, cavities, infiltrates, DAH Kidney – higher eGFR at presentation, more active lesions (necrosis and crescents) Heart – rare involved, often asymptomatic ANCA-more often, but not exclusively associated with PR3 Histology – granulomatous inflammation, vasculitis	Typically lung and kidney involvement ENT – considered to be rare Lungs – pulmonary fibrosis, DAH Kidney – more chronic lesions (sclerosis and tubulointerstitial fibrosis) Heart –uncommon ANCA-more often, but not exclusively associated with MPO Histology - only vasculitis, lacking of granulomatous inflammation	Associated with eosinophilia, asthma is a predominant respiratory symptom ENT – allergic symptoms, non-destructive Lungs – infiltrates and nodules, DAH rarely seen but also may occur Kidney – occurs less likely and a lesser degree Heart – often involved and associated with damage and higher mortality ANCA – mainly MPO, but detected only in about 40% of patients Histology – granulomatous inflammation usually accompanied by eosinophils, eosinophilic vasculitis

GPA, granulomatosis with polyangiitis; MPA, microscopic polyangiitis; EGPA, eosinophilic granulomatosis with polyangiitis; ENT, ear, nose, throat; DAH, diffuse alveolar hemorrhage; ANCA, antineutrophil cytoplasmic antibodies; MPO, myeloperoxidase; PR3, proteinase 3, eGFR; estimated glomerular filtration rate.

### 4.4 Phenotypes

In the last decade, several studies have indicated that ANCA specificity better characterized AAV patients, than clinical diagnoses of GPA or MPA, regarding to clinical features, treatment response, and prognosis (134, 135). Moreover, the rationale for an ANCA serology-based subclassification was further supported by evidence of genetic susceptibility (14), and immunological profile (136). Generally, patients with PR3-ANCA positivity more often have a presentation consistent with GPA, whereas those with MPO-ANCA tend to have features of MPA [however, about 10% of GPA patients are MPO-ANCA positive, and in MPA, PR3-ANCA can be also detected (137)]. Consistently, in AAV with PR3-ANCA positivity granulomatous pattern dominates, while in AAV with MPO-ANCA, vasculitic pattern is predominantly present (138). Given the significant overlap in clinical presentations and the separate genetic associations, reclassification of AAV is being considered according to the ANCA type (PR3 versus MPO) rather than traditional clinical phenotype (GPA versus MPA).

The combination of ANCA type with clinical phenotype identifies additional subtypes with unique features. Several studies have shown that MPO-ANCA positive GPA patients have more frequently limited disease, and a high prevalence of SGS, as well as fewer constitutional symptoms and milder renal lesion at diagnosis compared with those tested as PR3-ANCA positive. In addition, they are predominantly female and younger, and have significantly lower relapse rates (134, 137). However, in the recent study, within the GPA group (N = 151, 29% MPO+) patients with MPO-ANCA were older, and characterized by less prevalent ENT and neurological manifestations, increased end-stage renal disease (ESRD) and mortality (139).

In part of patients with AAV, co-presentation of ANCA and anti-GBM antibody occurs, what is associated with distinguish phenotype. This dualism affects predominantly patients with MPO-AAV (10–40%), while among those with PR3-AAV about 5–14% have circulating anti-GBM (3). Double-positive patients share characteristics of AAV, such as older age and longer symptom duration before diagnosis, and features of anti-GBM disease, such as severe renal disease and high frequency of lung hemorrhage at presentation (140).

Finally, recently three subclasses of AAV have been proposed, based on ANCA specificity during it clinical course, and described as non-severe AAV (mainly PR3-AAV limited disease), severe PR3-AAV (with multiorgan potentially life-threatening manifestation), and severe MPO-AVV (with dominant symptoms of vasculitis and chronic organ damage) (138). More recently, the new fourth subphenotype has been identified based on serological (including ANCA type) and clinical features, characterized by multiorgan involvement, high risk relapse, relatively young age, and marked mortality (141).

Interestingly, there are preliminary studies reported the association between clinical characteristics and specific peripheral immunological profile in patients with AAV allowing classify these patients into originally useful subgroups associated with specific organ involvement (CNS and kidneys) and defined therapeutic prognosis. These promising results suggest that immuno-phenotyping may become a new way to better identify and classify patients with AAV in the future (142).

## 5 Disease assessment and prediction of relapse

### 5.1 Severity of the disease

After diagnosis, GPA should be classified according to its extent and severity. There have been several methods to grade disease severity in patients with GPA (Table 5) (68, 73, 143–145); however, the most recent EULAR recommendations maintained the AAV categorization proposed in 2016 that distinguishes patients with and without organ-threatening or life-threatening disease, instead into those with “severe” and “non-severe” AAV, with the classification of the organ symptom as life-threatening or not, depends on the clinical assessment of individual patients (73). In 1996, the FVSG created the Five Factor Score (FFS), a prognostic score for patients with PAN, EGPA and MPA associated with severe organ involvement (146), while revised in 2009 (published in 2011) included all types of small vessel vasculitis, with the addition of GPA particularly. This scoring system distinguishes AAV with good (FFS = 0) or bad (FFS ≥ 1) prognosis with the sole criterion of measuring mortality (68).

**Table 5:** Assessment of extension and severity of AAV (including GPA).

WGET Research Group 2002	EUVAS 2009	FVSG (FFS) 2009	ACR 2021		EULAR 2022	
			Severe disease	Non-severe disease	Disease with organ/life-threatening manifestations	Disease without organ/life-threatening manifestations
1.Limited -disease which do not pose an immediate threat to either a critical organ or to the patient’s life, specifically: - There are not RBC casts in the urine - The serum Cr must be ≤1.4 mg/dL and the rise in serum Cr cannot be greater than 25% above the patient’s baseline - Lug involvement must be circumscribed (the room air Pa0^2^ > 70 mmHg or air 0^2^ saturation by pulse oximetry >90%) - No disease may exist within any other critical organ (e.g., GI, eyes, CNS) 2. Severe – disease which is not classifiable as limited	1. Localized – limited to the upper and/or lower respiratory tract 2. Early systemic-any, without organ-threatening disease 3. Generalised-renal or other organ-threatening disease, Cr < 500 μmol/L (5.6 mg/dL) 4. Severe – renal or other vital organ failure, Cr > 500 μmol/L (5.6 mg/dL) 5. Refractory – progressive disease unresponsive to treatment	1.Age > 65 years 2.Cardiac insufficiency 3.Renal insufficiency (stabilized peak Cr ≥ 150 μmol/L) 4.GI 5.Absence of ENT FFS = 0 non-severe disease FFS ≥ 1 severe disease Mortality rates: FFS = 0 9% FFS = 1 21% FFS ≥ 2 40%	Vasculitis with life- or organ-threatening manifestations, e.g., DAH, glomerulonephritis, CNS involvement, mononeuritis multiplex, cardiac involvement, mesenteric ischemia, limb/digit ischemia	Vasculitis without life- or organ-threatening manifestations, e.g., rhinosinusitis, mild systemic symptoms, uncomplicated cutaneous disease, mild inflammatory arthritis	DAH Glomerulonephritis Meningeal involvement CNS involvement Retro-orbital disease Cardiac involvement Mesenteric involvement Mononeuritis multiplex	Nasal and paranasal disease (without bony erosion, cartilage collapse or olfactory dysfunction or deafness) Skin involvement (without ulceration) Myositis (skeletal muscle only) Non-cavitating lung nodules Episcleritis
					There are only examples (many other symptoms of AAV exist); assessment of severity may differ in the individual patients	

FFS, five-factor score; GPA, granulomatosis with polyangiitis; GI, gastrointestinal involvement; Cr, creatinine; RBC, red blood cells; CNS, central nervous system; ENT, ear, nose, throat; DAH, diffuse alveolar haemorrhage; ACR, American College of Rheumatology; FVSG, French vasculitis study group; EULAR, European league against rheumatism; EUVAS, European vasculitis study group; WGET, Wegener’s granulomatosis etanercept trial.

### 5.2 Disease activity and monitoring

#### 5.2.1 Laboratory markers

Due to the chronic nature, the tight follow-up of GPA is necessary to provide an appropriate management, however, for date, no reliable marker for disease activity or to predict relapse has been found (147–150). Basic inflammatory markers, as ESR and CRP are non-specific with limited clinical use (151); however, some studies demonstrated that when a combined rise in CRP, neutrophil count, and PR3-ANCA was observed in the 6-month period before a relapse event, 59% of patient relapses could be predicted (152). The new intensively developing technology is metabolomics analysis exploring metabolic changes in AAV patients well separating these entities from other diseases (153). Furthermore, in recent study, in patients with PR3-AAV, homozygosity for PRTN3-Val^119^Ile polymorphism appears associated with higher frequency of severe relapse. However, further studies are necessary to better understand the association of this observation with the risk of severe relapse (154).

Regarding the ANCA, the value of its testing in GPA monitoring remains controversial and still is being debated (135, 155–159). Some studies indicated that patients achieving ANCA negativity during remission are 40% less likely to relapse (160), while those with persistent ANCA have higher risk of relapse, with a higher rate among positive PR3 patients with renal involvement or DAH treated with rituximab (57). However, in other studies, decreases in PR3-ANCA levels were not associated with shorter time to remission, and increases were not associated with relapse (158, 161). This discordance between ANCA serology and GPA activity limits support for its use as a reliable biomarker. However, despite these discrepancies, results of recent clinical studies demonstrated that in patients PR3-ANCA positive treated with rituximab, relapses without at least one event between B-cells repopulation or rise of ANCA titers were unusual, what could indicate that combining use of these two biomarkers may be value to predict relapse, especially in this specific cohort (159). Nevertheless, it is not currently recommended to perform ANCA testing for GPA monitoring and the sole increase in ANCA concentrations or reappearance of ANCA is not an indication for treatment implementation, if it is not accompanied by clinical symptoms (73, 76).

**Figure 4:**
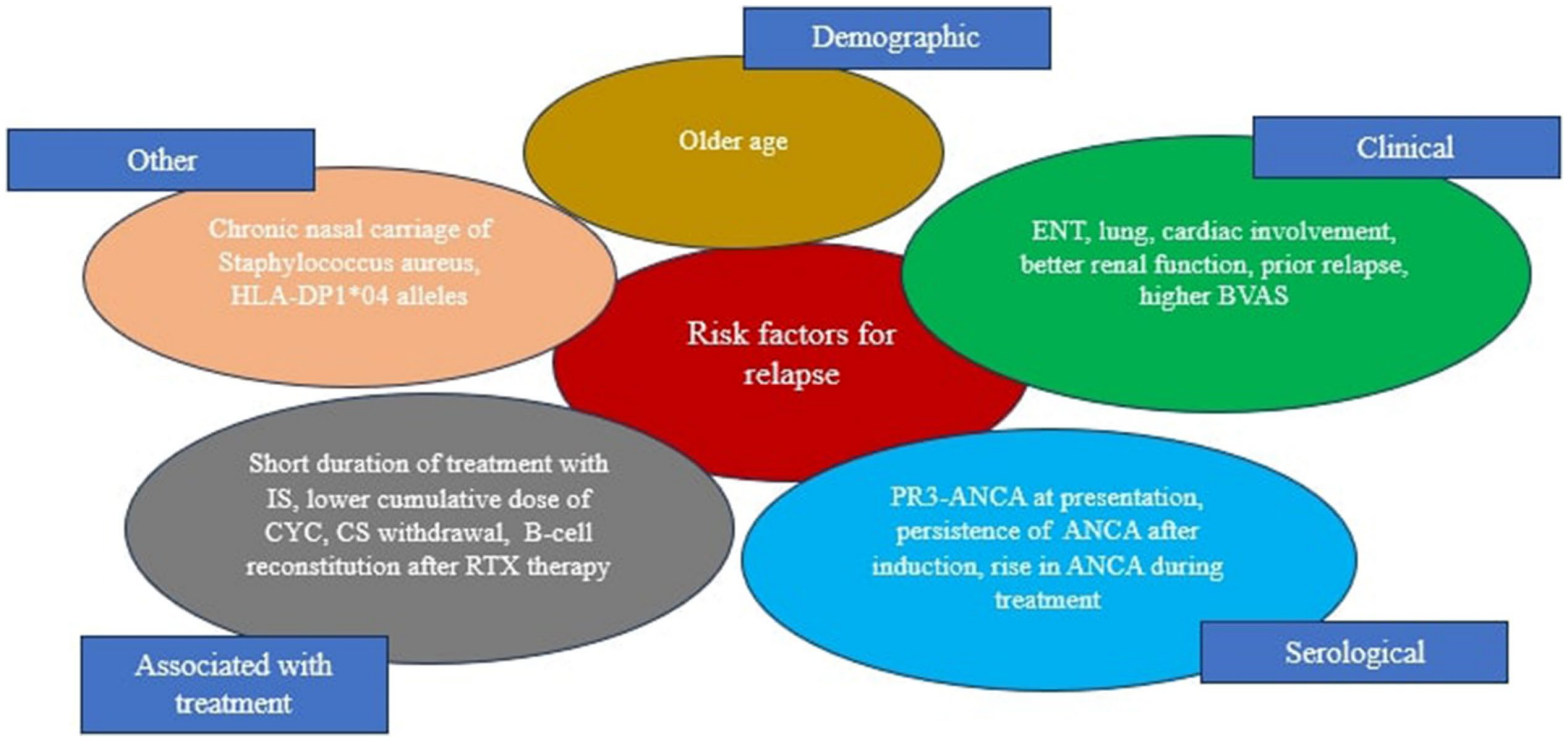
Factors increasing the risk of relapse of AAV.

Although we still do not have sufficient knowledge how to predict relapse of GPA, some clinical and serological features associated with higher risk of relapse have been reported (156, 159, 162) (Figure 4). More recently, the FVSG has been proposed and validated a relapse scoring system for AAV patients based on the PR3-ANCA positivity, older age (≥ 75 years), and higher eGFR (≥ 30 mL/min/1.73 m^2^). Each of these items was assigned one point (range 0–3), with the 5-year relapse risk at 8% for zero points, 30% for one point, 48% for two points, and 76% for three points in the validated cohort (63). This score may be used in clinical practice, however its significant to tailored duration of maintenance therapy requires further prospective studies.

#### 5.2.2 Clinical assessment

While the search for a marker of disease activity is still ongoing, the structural clinical patient evaluation is recommended (73), with the Birmingham Vasculitis Activity Score (BVAS) is an approved, easily available, and effective tool to evaluate disease activity in clinical practice (163). BVAS has negative prognostic value (164) and it has been identified as predictive factor for relapse (162) and a risk factor for venous thromboembolism in patients with AAV (165). Interestingly, patients with AAV who were ever smoked have increased BVAS and more frequent renal replacement therapy and IS treatment, resulting in a poorer survival prognosis (166). The next clinical score system designed and validated for use in patients with GPA is the Disease Extent Index (DEI), but it involves only symptoms attributable to active vasculitis (not persistent symptoms) (167). This system correlates well with the BVAS, however, it quantifies different domains of the disease, and some authors recommend its use in conjunction with the BVAS in assessments of patients with GPA (167). Finally, the Vasculitis Damage Index (VDI) is a sensitive and credible clinical tool for quantifying damage in patients with AAV (168), however, it should be well differentiated from active vasculitis. VDI has been identified as one of the factors associated with higher mortality in AAV patients (169). In addition, although VDI and BVAS are separate systems scores, the link between them has been demonstrated. In the recent study analyzing the prevalence and impact on damage accrual of different levels of disease activity in AAV patients, the prolonged low disease activity state (defined as 0 < BVAS ≤3 and taking low dose of CS ≤ 7.5 mg/day and lasting ≥2 consecutive years) correlated with increased VDI (170).

As AAV has progressed from a life-threatening disease to a chronic, relapsing condition, the importance of assessing the disease from the patients’ perspective has become increasingly important. The Outcome Measures in Rheumatology (OMERACT) Vasculitis Working Group drew attention to this issue and proposed a composite assessment tool for AAV based on the BVAS, VDI, mortality and including patient reported outcomes (PROs) (171). More recently, an international survey has been conducted (Delphi exercise) aimed to reach consensus about which measures are considered by patients and physicians to be most important when assessing activity of AAV (172). The consensus between patients and physicians on many items has been found, however, achievement of specific BVAS scores were highly rated only by physicians, while items highly rated only by patients included laboratory measures (changes on urinalysis and acute phase reactants), pain and fatigue. These data indicates that patients’ participation in the assessment of disease activity may increase significantly in the future.

## 6 Conclusion

Major developments in the diagnosis of GPA have been made over the past decades. New imaging techniques allow recognition of specific organ involvement, and the new classification criteria contributes to better qualify the patients for the epidemiological and therapeutic studies. However, the needs such as the lack of uniformity and validated diagnostic criteria remain unmet, and the question is, whether and how the current classification criteria could help to develop the diagnostic criteria to be translated into clinical practice (173). Furthermore, a reliable biomarker to predict relapse is still lacking, which poses a risk of suboptimal disease control or unnecessary patient exposure to potentially toxic therapy (151). A big step forward is distinguishing new AAV phenotypes based on the ANCA specificity and significant advances in identifying the nuances between PR3-ANCA and MPO-ANCA vasculitis. In turn, the increasing knowledge about the distinct immunological profiles may provide an understanding disease pathophysiology and prognosis, with the result that immuno-phenotyping may become a new way to better identify patients with AAV in the future (142).

## Ethics statement

Written informed consent was obtained from the individual(s) for the publication of any potentially identifiable images or data included in this article.

## Author contributions

MP-P: Writing – original draft. JF: Conceptualization, Supervision, Writing – original draft, Writing – review & editing.
